# Knowledge and Attitudes Regarding Human Papillomavirus Vaccination Among Future Healthcare Workers in Serbia

**DOI:** 10.3390/vaccines13010011

**Published:** 2024-12-26

**Authors:** Nemanja Maletin, Nikola Denda, Ana Ljubičić, Radmila Velicki, Aleksandra Patić, Zoran Golušin, Tihomir Dugandžija, Vladimir Petrović, Mioljub Ristić, Vladimir Vuković

**Affiliations:** 1 Faculty of Medicine, University of Novi Sad, 21000 Novi Sad, Serbia; 910070d24@mf.uns.ac.rs (N.M.); 902018d24@mf.uns.ac.rs (N.D.); radmila.velicki@mf.uns.ac.rs (R.V.); aleksandra.patic@mf.uns.ac.rs (A.P.); zoran.golusin@mf.uns.ac.rs (Z.G.); tihomir.dugandzija@mf.uns.ac.rs (T.D.); vladimir.petrovic@izjzv.org.rs (V.P.); mioljub.ristic@mf.uns.ac.rs (M.R.); 2 Institute of Public Health of Vojvodina, 21000 Novi Sad, Serbia; ana.ljubicic@izjzv.org.rs; 3 Clinic for Dermatovenerology, University Clinical Center of Vojvodina, 21000 Novi Sad, Serbia; 4 Oncology Institute of Vojvodina, 21204 Sremska Kamenica, Serbia

**Keywords:** human papillomavirus, HPV vaccination, knowledge and attitudes, prevention, students

## Abstract

Background/Objectives: Adequate knowledge and correct attitudes about the HPV vaccine influence awareness of the importance of preventing HPV-related diseases, which is particularly important for future healthcare professionals. We aim to examine the share of correct answers and the prevalence of different attitudes about the HPV vaccine among active regular students of the Faculty of Medicine in Novi Sad. Methods: A cross-sectional study was conducted from 1 to 30 November 2023 using a structured survey questionnaire. Results: A total of 1760 students were included, of which 78% were female, with an average age of 21 years. Students who participated in prior HPV education) demonstrated significantly higher knowledge (81.92% vs. 65.60%, *p* < 0.001) and were more likely to recommend the vaccine to patients (89.91% vs. 82.99%, *p* < 0.001). Almost all vaccinated students (99.41%) would recommend the vaccine, compared to 82.91% of unvaccinated students (*p* < 0.001). Students who actively sought HPV information also showed a higher likelihood of recommending the vaccine (93.05% vs. 83.02%, *p* < 0.001). Moreover, those with sufficient self-assessed knowledge were more inclined to recommend the vaccine (89.88%) than those with insufficient knowledge (81.66%, *p* < 0.001). The analysis demonstrated that an increase in the number of correct answers in the knowledge evaluation corresponds to higher odds of recommending the HPV vaccine to patients (OR = 1.23, 95% CI 1.17–1.28). Positive attitudes prevailed, with 68.89% supporting more education on HPV vaccination. Conclusions: Students who previously attended education on HPV infection/vaccination and those who would recommend the vaccine have significantly higher levels of knowledge. The study highlights the importance of HPV-related education in shaping future healthcare professionals’ attitudes and knowledge.

## 1. Introduction

Human papillomavirus (HPV) infection is one of the most common sexually transmitted infections in the world [1]. Most sexually active women and men will be infected with at least one type of HPV during their lifetime [2]. In most cases, HPV will be eliminated by acquired immunity and will not lead to a manifested disease [3]. In a minority of cases, it causes diseases ranging from asymptomatic infections to pre-cancers and cancers. The peak incidence of HPV infection occurs between the ages of 20 and 25, after which it declines, reaching a plateau around the age of 35 [4]. A high prevalence of HPV infection has been observed in less developed countries [4].

A better understanding of HPV biology, its effect on human cells, and immune system reactions to its presence has enabled the development of an effective prophylactic vaccine against HPV. Reduction in the prevalence and incidence of HPV infection and, consequently, genital warts and cervical lesions has been observed in regions with good HPV vaccination coverage [3]. Previous research has shown that when the vaccine is used in HPV-negative young women under 25 years old in a three-dose regimen, the efficacy of HPV vaccines is approximately 100% [5].

Cervical cancer remains a leading cancer among women in Serbia, where in the previous period, the incidence was demonstrated to be higher than the European average [6]. Thus, it is important to achieve optimal vaccination coverage to protect a large part of the population from HPV types contained in the vaccine. In the Republic of Serbia, the quadrivalent HPV vaccine has been available on the private market since 2006; however, the coverage rate for HPV immunization remained low. Since 2022, the quadrivalent HPV vaccine has been replaced by the nine-valent, which is now provided free of charge. The vaccine is administered in two doses for children aged 9 to 14 and in three doses for individuals aged 15 and older [7].

Immunization coverage in Serbia during the first year of the vaccination programme, free of charge, was low, i.e., 5.5% in the cohort of girls 9–14 years old and 5.9% among those aged 15–19 years [8]. Sufficient knowledge and a positive attitude towards HPV vaccination in the population are needed to achieve optimal vaccination coverage. A recent Italian cross-sectional study among adults explored the reasons for receiving the HPV vaccine and underlined that multidisciplinary efforts, through tailored educational programmes and health interventions, are needed not only to increase the level of knowledge but also to promote positive attitudes about the HPV vaccine [9]. Other studies have also shown the importance of campaigns to raise knowledge about HPV and awareness of vaccination, especially targeting young people and students [3,5,10]. Considering these findings and the most commonly cited reasons for rejecting the HPV vaccine, it is important to emphasize the powerful impact that motivated and well-informed healthcare workers can have on increasing vaccination rates [11]. Their active engagement and advocacy, combined with the support from personal networks, can play a key role in overcoming public hesitations and ensuring broader acceptance of the vaccine. Proper education of future healthcare professionals about HPV infection and vaccination is crucial to forming correct attitudes and acquiring adequate knowledge so that they are well prepared to provide effective vaccine recommendations and ensure the best possible health advice for their patients [12,13].

This study aimed to analyze various aspects of knowledge and attitudes regarding HPV vaccination among different study profiles of students, i.e., future healthcare workers.

## 2. Materials and Methods

### 2.1. Study Design, Participants, and Sampling

This cross-sectional study was conducted from 1 to 30 November 2023 at the Faculty of Medicine, University of Novi Sad (MFNS), Novi Sad, Serbia, among undergraduate students of all study programmes conducted in Serbian language. The faculty offers a diverse range of study programmes in the Serbian language, including medicine, pharmacy, dentistry, nursing, medical rehabilitation, radiological technology, and special education and rehabilitation, playing a crucial role in the training of future healthcare professionals in the country. The MFNS is the second largest medical faculty in Serbia, with approximately 5000 enrolled students (4235 enrolled in study programmes in Serbian and 673 foreign students attending classes in English). Sample size was determined based on the total number of actively attending students at the MFNS, in the academic year 2023/24. Of 4235 enrolled students, there were 2513 active regularly attending classes in the academic year 2023/24, which were considered eligible to participate. A total of 1760 active students participated in this study (the response rate was approximately 70% of the regularly attending students in the academic year 2023/24). Having 2513 active regularly attending classes, we calculated the enrolment of at least 334 participants required to achieve the 95% level of confidence. We considered the non-response correction of 10% and arrived at the sample size of 368 participants, giving us a good chance of detecting the true difference between the explored variables, where it exists.

Exclusion criteria for this study included students who were not actively attending classes during the academic year 2023/24, foreign students enrolled in the English-taught study programmes, and those who provided incomplete survey responses (more than 50% of the missing answers). The study was designed and conducted following the recommendations from the Consensus-Based Checklist for Reporting of Survey Studies (CROSS) [14], and detailed specification of the reported items is presented in Supplementary Table S1.

**Figure 1:**
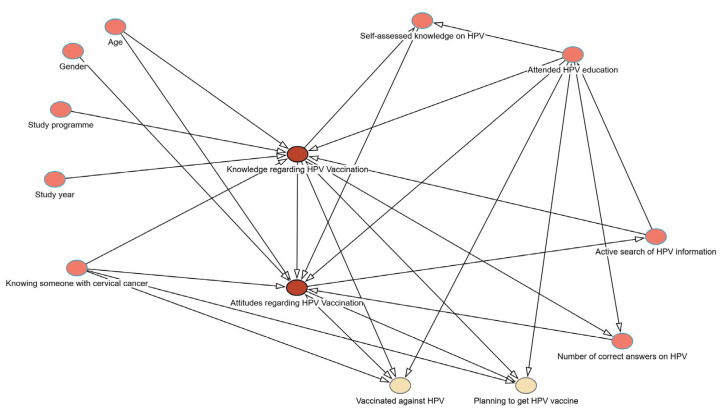
Conceptual framework of the study using directed acyclic graph (DAG). Note: Arrows represent the direction of the potential effects of one variable on another.

A conceptual framework based on the available literature and study design features, explaining the potential effects of one variable on another and their relationship with the knowledge and attitudes regarding HPV vaccination is created using directed acyclic graph (DAG) analysis (DAGitty version 3.1.) [15] and is presented in Figure 1.

### 2.2. Data Collection

Data were collected using a survey questionnaire in Serbian language (the English version is presented in Supplementary Table S2). Prior to the start of the survey, a brief announcement detailing the study and its starting date was posted on the faculty notice board and disseminated among students. Recruitment was carried out in coordination with teaching staff, where recruiters randomly visited classrooms before the start of lectures. They distributed questionnaires to all students actively attending classes during November 2023. This process was repeated throughout the month to ensure that all groups, study programmes, and study years at the MFNS had the opportunity to participate in the survey during November 2023. Initially, a pilot study was conducted on 20 participants to assess the comprehensiveness of the questionnaire and the clarity of the questions and answers. The first part of the questionnaire contained 16 questions related to socio-demographic characteristics, previous education on HPV vaccination, subjective assessment of knowledge, and HPV vaccination status. The second part of the questionnaire contained 11 statements about the HPV vaccine to assess the respondents’ knowledge about the HPV vaccine and vaccination. Each item in the questionnaire had three possible response options: affirmative (Yes), negative (No), and uncertain (I don’t know). The third part of the questionnaire was used to assess attitudes about HPV vaccination and included nine selected statements for which the respondent could rate their level of agreement from 1 to 5 (1 completely disagree, to 5 completely agree).

### 2.3. Ethical Considerations

The study protocol was approved by the Ethics Committee of the Faculty of Medicine, University of Novi Sad (approval number: 01-39/97/1, from 31 October 2023). Participation in this research was voluntary, anonymous, and without material compensation. Respondent gave their written consent to participate. Each respondent was assigned a unique code, after which all data were entered into a database on a computer with protected access limited to members of the research team.

### 2.4. Statistical Analysis

We used descriptive statistics, and data were presented as mean with standard deviation (SD) or as frequencies and proportions. Participants were categorized into three groups based on their age, i.e., 18–19 years old since they were eligible to receive HPV vaccine free of charge; 20–25 years old as the range of years considering participating in the longest study programme (i.e., 6 years for medicine); and >25 years old. Also, we grouped students from study programmes into two groups—health professions that can directly promote HPV vaccination, i.e., study programme of medicine and nursing and pharmacy (group 1), and health professions that can indirectly promote HPV, i.e., not directly working with patients/parents interested in HPV vaccination but can informally promote it, like study programme of dentistry, medical rehabilitation, radiologic technology, special education, and rehabilitation (group 2). Finally, we separately analyzed the answers of students enrolled in study programmes that had the chance to attend subjects with lessons on HPV infection/vaccination during regular classes in epidemiology, pediatrics, and gynecology, i.e., sixth-year medicine and third and fourth years of nursing, versus all other students.

For the comparison of continuous variables, independent samples t-test or Wilcoxon rank-sum test were used, depending on the assessment of normality of distribution. For comparing differences in the distribution of categorical variables, the Chi-square test was used. Univariate and multivariate linear regression analysis was used to examine potential predictors of the willingness of healthcare students to recommend the HPV vaccine to their future patients. Throughout the analyses, *p* < 0.05 (two-tailed) was considered as statistically significant. All statistical analyses were performed using the statistical software STATA v.17 (College Station, TX, USA: StataCorp LLC 2021).

## 3. Results

**Figure 2:**
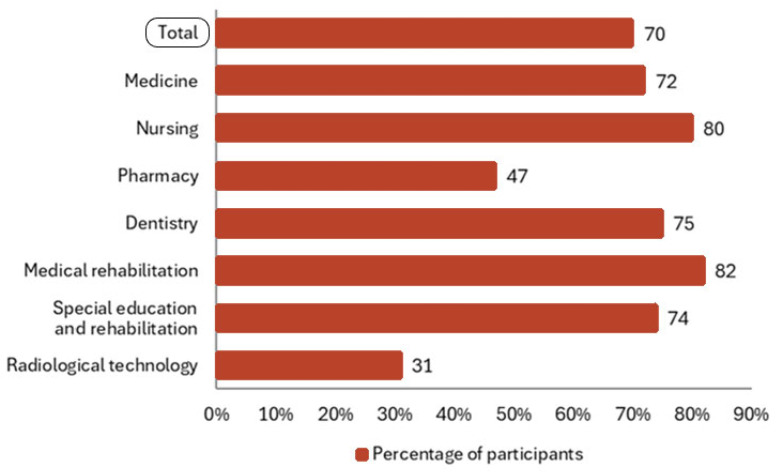
Percentage of the included participants by each study programme of the MFNS.

Around 2500 questionnaires were distributed to all active students of the MFNS attending classes in the academic year 2023/24, and 1760 completed questionnaires were collected and included in the study, representing 70% of all actively enrolled students at MFNS in the academic year. The highest percentage of completed questionnaires was among students of the medical rehabilitation study programme (82%), followed by students of nursing (80%), dentistry (75%) and special education and rehabilitation (74%) programmes, while the lowest percentage was collected from the radiologic technology programme (31%) as presented in Figure 2.

A total of 1178 (67%) students were in group 1, and 582 (33%) were in group 2, while group 1 has a slightly higher share of students over the age of 25 (7.56% vs. 2.41%). A higher percentage of students from group 2 (52.32%) do not plan to get vaccinated, while this percentage is lower in group 1 (44.21%, *p* = 0.004). Students from group 1 significantly more often attended HPV education sessions (29.54%) compared to students from group 2 (17.53%, *p* < 0.001), and students from group 1 more often actively sought information about HPV (17.57% vs. 9.11%, *p* < 0.001). Additionally, students from group 1 more frequently assess their knowledge of the HPV vaccine as sufficient (38.88%) compared to students from group 2 (31.10%, *p* = 0.002). Detailed socio-demographic characteristics are presented in Table 1.

Also, we separately analyzed answers of students enrolled in study programmes that had subjects with lessons on HPV infection/vaccination during regular classes in epidemiology, pediatrics, and gynecology classes, i.e., sixth-year medicine and third and fourth years of nursing, versus all other students and the results remained highly significant (Supplementary Table S2). There was a higher percentage of students who self-assessed their knowledge as sufficient (60.8%), those who would recommend a vaccine to a future patient (95.48%) and to a friend/family member (90.95%) in the group those who attended classes with HPV-related topic (*p* < 0.001, *p* < 0.001, and *p* < 0.001, respectively).

**Figure 3:**
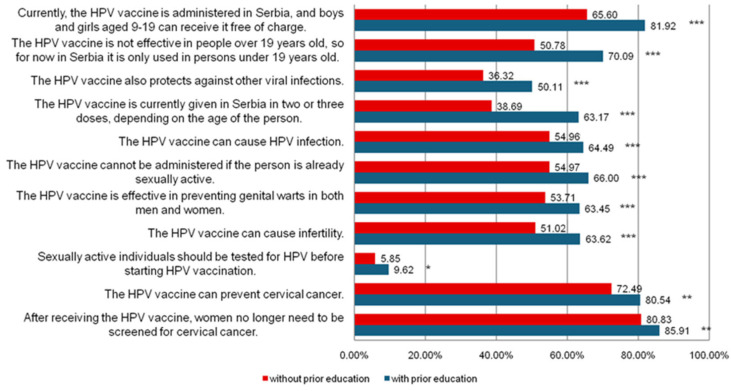
Share of correct answers in relation to prior education on HPV infection/vaccination. Note: Using Chi-square test; * *p* = 0.002; ** *p* = 0.001; *** *p* < 0.001.

Figure 3 shows that students who attended prior education on HPV have a higher share of correct answers compared to those who did not attend prior education, i.e., 81.92% of students who attended prior education know that the HPV vaccine is given free of charge to children in Serbia, while only 65.60% of students who did not attend prior education are aware of this. Only 36.32% of students without prior education correctly answered the statement that the HPV vaccine also protects against other viruses, while students with prior education had a significantly higher percentage of correct answers (70.09%). Additionally, they are less likely to believe in incorrect information, such as the claim that the HPV vaccine causes HPV infection (64.49% with prior education vs. 54.96% without prior education, *p* = 0.001) or that the vaccine can cause infertility (63.62% vs. 51.02%, *p* = 0.001). The smallest percentage of students answered correctly to the statement about HPV testing before HPV vaccination, but there is still a higher percentage of correct answers among students who attended education (9.62% vs. 5.85%, *p* = 0.002).

The results in Table 2 show that students who attended HPV education in the past year are significantly more likely to recommend the HPV vaccine to their future patients. Specifically, 89.91% of students who attended education would recommend the vaccine, while the percentage is lower among those who did not (82.99%, *p* < 0.001). Similar was for those who attended the study programme with lessons on HPV infection/vaccination in comparison to those who did not (95.96% vs. 83.15%, *p* < 0.001). Nearly all vaccinated students (99.41%) would recommend the vaccine, whereas the figure is lower among the unvaccinated (82.91%, *p* < 0.001). Additionally, students who actively sought information about HPV and vaccination in the past month are significantly more likely to recommend the vaccine (93.05%) compared to those who did not seek such information (83.02%, *p* < 0.001). Students who believe they have sufficient knowledge about the HPV vaccine are more likely to recommend it to their future patients (89.88%) than those who feel they lack sufficient knowledge (81.66%, *p* < 0.001).

Additionally, students who attended HPV education in the past year would significantly more often recommend the HPV vaccine to family members or friends (86.39%) compared to those who did not attend education (81.14%, *p* = 0.012). Also, students of study programmes with classes on HPV infection/vaccination would in higher percent recommend vaccine (93.30%) with respect to students of other study programmes (81.07%) (*p* < 0.001). Similarly, almost all students who have been vaccinated with the HPV vaccine (99.40%) would recommend it, while this percentage is lower among unvaccinated students (80.50%, *p* < 0.001). Students who actively sought information about HPV and vaccination in the past month are also significantly more likely to recommend the vaccine (91.80%) compared to those who did not (80.61%, *p* < 0.001). Students who believe they have sufficient knowledge are more likely to recommend the vaccine to their family members and friends (87.00%) compared to those who believe they have insufficient knowledge about the HPV vaccine (79.87%, *p* < 0.001).

The results in Table 3 show that 72.5% of students who know that the HPV vaccine is given free of charge to boys and girls aged 9–19 in Serbia would recommend the vaccine to their patients, compared to 56.6% of those who would not recommend the vaccine (*p* = 0.001). The lowest percentage of correct answers was recorded for the statement about testing sexually active individuals for HPV before HPV vaccination (6.88%). Of the students who would recommend the HPV vaccine to their future patients, only 7.5% knew that testing for HPV is not required before vaccination (*p* = 0.001). Additionally, among the students who would recommend the vaccine to their family members and friends, only 7.6% knew that testing for HPV is not required before vaccination (*p* = 0.001). The results on the percentage of correct answers per respondent were presented as median with interquartile range (IQR). This shows that the median percentage of correct answers for those who recommend the vaccine to future patients is 63.64% (IQR 45.46–72.73), while for those who do not recommend the vaccine, it is lower at 45.46% (IQR 27.27–63.64). These differences are statistically significant, with a *p*-value of less than 0.001. A similar pattern is observed for recommendations to family members and friends.

**Figure 4:**
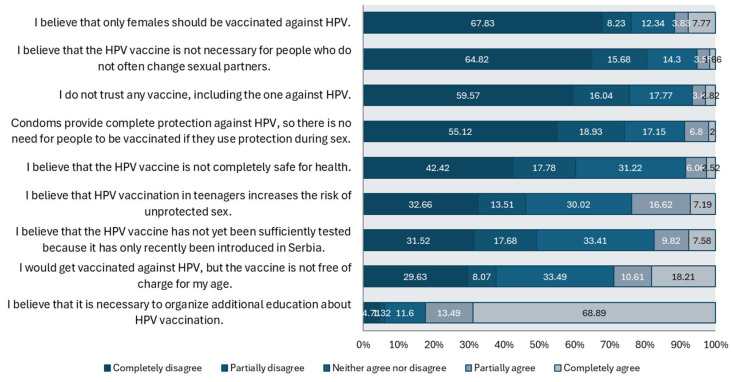
Prevalence of attitudes about the HPV vaccine among all participants.

By examining students’ attitudes, it was observed that the majority have correct views regarding HPV vaccination. Most students (67.83%) completely reject the statement that only women should be vaccinated against HPV. Additionally, 68.89% of students completely agree that it is necessary to organize additional education on HPV vaccination. On the other hand, 17.77% of students express distrust towards all vaccines, including the HPV vaccine, while 17.15% believe that condoms provide complete protection against HPV. Detailed results on students’ attitudes are presented in Figure 4.

The results in Supplementary Figure S1 indicate that students who attended HPV vaccination education have significantly more positive attitudes compared to those who did not. For example, 68.68% of students who attended the education disagree with the statement that the HPV vaccine is not necessary for people who do not often change sexual partners, compared to 63.52% of students who did not attend the education (*p* = 0.006). Trust in vaccines is also higher among students who attended the education, with 66.67% rejecting the claim that they do not trust any vaccine, including the HPV vaccine, compared to 57.06% of those who did not attend the education (*p* = 0.003). Additionally, a significantly higher percentage of students with education reject the incorrect statement that condoms provide complete protection against HPV (62.72% vs. 52.27%, *p* < 0.001).

When we compared the attitudes of students of the study programmes with regular classes on HPV infection/vaccination topic versus those without, we noticed that also here those with classes had positive attitudes in higher percentage (Supplementary Figure S2). Specifically, almost 81% of students completely disagree that they trust any vaccine, including HPV, in the group of students with classes on HPV-related topics in comparison to 56.93% of those in the other group (*p* < 0.001). Also, 68.7% of students after HPV-related classes completely disagree that condoms provide complete protection against HPV and that there is no need to be vaccinated if they use protection during sex, in respect to 53.41% of students from the other group (*p* < 0.001).

The results of univariate and multivariate logistic regression analysis, exploring the predictors of future healthcare workers’ willingness to recommend the HPV vaccine to their patients, are presented in Table 4. With each additional year of age, the odds of a positive outcome increase by 8% (OR = 1.08, 95% CI 1.001–1.15, *p* = 0.048). Fifth-year students have a significantly higher likelihood of recommending the vaccine in the unadjusted model (OR = 1.71, 95% CI 1.04–2.81, *p* = 0.033), which increases in Model 1 (OR = 2.38, 95% CI 1.11–5.12, *p* = 0.026), while sixth-year students show an even more pronounced trend with an OR of 8.89 in the unadjusted model and 12.53 in Model 1 (*p* < 0.001). Also, students who had the opportunity to attend regular classes with HPV-related topics had almost five times higher odds of recommending the vaccine with respect to other students (OR = 4.81, 95% CI 2.34–9.89, *p* < 0.001; aOR = 4.25, 95% CI 2.02–8.95, *p* < 0.001). Students who plan to get vaccinated have a significantly higher odds ratio (OR) for this outcome compared to those who do not plan to get vaccinated (OR = 16.06, 95% CI 8.13–31.73 in the unadjusted model, *p* < 0.001, OR = 17.19, 95% CI 8.63–34.23 in Model 1, *p* < 0.001). Similar results were found for students who would get vaccinated if the vaccine were free for their age group. Additionally, students who actively sought information about HPV have significantly higher odds of recommending the vaccine to their future patients compared to those who did not seek information (OR = 2.74, 95% CI 1.66–4.51 in the unadjusted model, *p* < 0.001, and OR = 2.48, 95% CI 1.50–4.11 in Model 1, *p* < 0.001). Finally, the greater the number of correct answers in the knowledge evaluation, the higher the odds of recommending the HPV vaccine to their patients (OR = 1.23, 95% CI 1.17–1.28 in the unadjusted model, *p* < 0.001, and OR = 1.19, 95% CI 1.14–1.25 in Model 1, *p* < 0.001).

## 4. Discussion

In this study, we thoroughly examined the knowledge and attitudes about the HPV vaccine as well as the HPV vaccination status among students of a medical faculty. Of the total number of enrolled students from all study programmes and years, about 70% participated in our research. Students who previously attended some kind of education on HPV infection/vaccination had significantly higher knowledge about the HPV vaccine and would, in a significantly higher percentage, recommend the vaccine to their patients as well as to their family members and friends, in respect to those that did not participate in such education. Examining the attitudes of students from all years and study programmes was observed that the majority of them have positive attitudes regarding the HPV vaccination.

The HPV vaccine is unique in its safety and, above all, its efficacy in preventing up to six types of cancer. In order to increase the uptake of HPV vaccine in our region, it is essential to maximize the use of all available resources, with highly motivated healthcare workers playing a crucial role alongside the support of networks of friends, family, and acquaintances [10]. By leveraging these influences and addressing specific barriers, such as fear of side effects, the perception that the vaccine is new or experimental, the belief that the risk of infection is low, and concerns about safety and insufficient information, we can significantly improve the effectiveness and sustainability of immunization programmes [16]. A recent systematic review investigated healthcare professional’s promotional strategies aimed at improving the HPV vaccine uptake in adolescents and concluded that healthcare professionals need to be better educated on the HPV vaccine in order to minimize their own hesitancy. Also, they suggested that additional training for healthcare professionals and better communication skills and tools, engagement, and supportive information can improve HPV vaccine uptake [17]. Similarly, a French study on the knowledge, attitudes, and practises of pharmacists regarding HPV infection and vaccination highlighted the critical role of healthcare workers in promoting vaccination and emphasized the need for additional education, effective communication tools, and supportive informational materials to reduce their hesitancy and improve the acceptance of HPV vaccination [18]. Together, these findings underline the importance of enhancing healthcare workers’ knowledge and equipping them with the necessary resources to actively advocate for and facilitate HPV vaccination, aligning with the observations from our study.

The relatively low vaccination rate among students in our study can be attributed to the fact that, although the HPV vaccine has been available in the Republic of Serbia since 2006, it only became free of charge in 2022 (one year after this study was conducted) and is currently offered without cost only to individuals aged 9 to 19 [10]. Compared to other similar studies conducted on student populations, our study recorded a lower percentage of vaccinated individuals in this population [19,20] but higher than the study from Turkiye, where only 3.5% of respondents had previously been vaccinated against HPV [21]. As explained by the authors, the result may be due to economic factors as well as insufficient awareness of HPV infection and the importance of HPV vaccination [21]. Students or future healthcare workers may have insufficient access to information about the benefits of HPV vaccination over the course of official educational programmes. According to another study from Hong Kong, family doctors should take on a more prominent role in disseminating accurate and precise information about HPV vaccination, emphasizing awareness of the risks associated with HPV as a sexually transmitted disease, as well as the availability of subsidized and safe HPV vaccines in tertiary educational institutions [22]. Such an approach could contribute to reducing barriers to vaccine acceptance and increasing vaccination coverage.

In our surveyed population, only 20% are motivated to get vaccinated against HPV in the future, with an additional 30% of respondents who would receive the vaccine if it was free of charge for their age group, which, compared to another research, is much lower [13,23]. As the vaccine is currently registered for persons up to 46 years old, a relatively high percentage of young adults in our study (47%) do not plan to get vaccinated in the future. Current and future primary healthcare providers should strongly endorse HPV vaccine recommendations and advocate vaccine acceptance to parents of children and adolescents [24,25]. For healthcare workers to recommend the HPV vaccine, they need to have sufficient knowledge and positive attitudes to personally and professionally influence the motivation for vaccination and consequently increase the number of vaccinated individuals. Even though, in our participants, we noticed the absence of risk perception in a situation when a person knows someone who had cervical cancer, this is not a motive for recommending the HPV vaccine.

About 26% of students in our sample attended some form of education on HPV infection and vaccination, with a statistically significant difference in responses between students who attended education and those who did not. It is crucial to increase awareness of HPV infection and vaccination as an effort to expand immunization coverage because awareness is an essential step in vaccination, and the method of increasing awareness is education [26]. As future healthcare providers, it is crucial for medical faculty students to be thoroughly educated about HPV infection, vaccination, and the importance of healthcare providers’ recommendations. This is particularly important considering the proven efficacy of the vaccine and the significant role healthcare provider recommendations play in patients’ health behaviours [27]. Studies have shown that most adolescents who receive a recommendation for HPV vaccination from the medical staff participate in vaccination, with one study showing that individuals who received a doctor’s recommendation are 35 times more likely to be vaccinated against HPV [12,28,29]. Many current efforts to increase HPV vaccination focus on improving the effectiveness of recommendations and educating the general population, healthcare workers, and students [30,31]. This emphasizes the importance and significance of thorough education for future healthcare providers in an environment conducive to learning, such as schools and universities.

In our study, we examined the share of correct answers in relation to prior education on HPV infection and vaccination. Students who attended education on HPV infection and vaccination had significantly higher knowledge compared to those who did not. The lowest number of correct answers was recorded for the statement about the need for prior HPV testing, i.e., before starting HPV vaccination, with less than 10% of respondents answering correctly. Regarding the question of whether the HPV vaccine also protects against other viruses, less than 40% of respondents answered correctly that it does not. Although students believe that HPV testing is necessary before vaccination, official recommendations state that HPV testing is not required before vaccination and that the vaccine is effective even when given to individuals who have had sexual intercourse [32]. However, it is important for women to continue undergoing cervical cancer screening even after receiving the HPV vaccine because the vaccine does not protect against all existing HPV types (but only those contained in the vaccines) or other sexually transmitted diseases [32]. Considering these results from our study, it is possible to plan future education programmes, specifically on which segments of HPV infection and vaccination to pay additional attention to during future educational sessions.

Also, we found that significantly more students who attended education on HPV infection and vaccination would recommend the HPV vaccine to their patients, friends, and family. From this result, the significance of education is also evident. Students willing to recommend the vaccine had significantly higher knowledge about the HPV vaccine and vaccination compared to students who would not recommend the vaccine. Knowledge can be considered a prerequisite for informed decision-making, i.e., if participants have a better level of knowledge about HPV infection and vaccination, they are more likely to take the initiative for HPV vaccination and recommend the vaccine to their patients, family, and friends [19]. Interest in the vaccine plays a significant role in raising awareness about HPV infection and vaccination as well as forming correct attitudes about the HPV vaccine, leading to later vaccine recommendations [13,16,21,33]. Multiple studies have shown that healthcare professionals with greater knowledge about HPV infection and vaccination are more likely to recommend the HPV vaccine to their patients [34,35,36]. This correlation has also been observed among medical science students, where those with more knowledge are more likely to recommend the vaccine [37,38]. Our study confirms that the greater the knowledge future healthcare workers have, the more likely they are to recommend HPV vaccines to their patients. A study from China showed that positive attitudes towards the HPV vaccine were associated with a higher likelihood of recommending it to others and that students who recognized the benefits of the vaccine were more likely to provide vaccination recommendations [12]. Similarly, our study demonstrates that students most commonly hold positive attitudes regarding the HPV vaccine and vaccination. Considering that current students are future healthcare workers, it is crucial that they have sufficient knowledge and form correct positive attitudes about vaccination to confidently recommend the vaccine to their future patients in the appropriate manner. Despite the suboptimal percentage of vaccinated students in both groups, with and without prior HPV education, and those students unwilling to get vaccinated, the attitudes towards HPV vaccination were positive in the majority of students in our study. This result can be explained by the fact that the research was conducted among medical faculty students, and other international studies have shown that medical faculty students have higher levels of knowledge and more positive attitudes regarding vaccination compared to students from other faculties [39,40]. This is understandable and commendable for medical faculty students as they represent the future educators and informers of the general public.

The results of our study emphasize the need for long-term monitoring of knowledge, attitudes, and beliefs about HPV vaccination in the community to ensure high vaccine acceptance and programme success. The findings from this study provide tangible evidence for researchers, healthcare workers, and public health policymakers as an impetus to conduct systematic studies on all aspects of HPV to fill gaps in knowledge and form correct attitudes, especially among future healthcare workers. Most importantly, to improve knowledge and awareness about HPV, a systemic response from relevant institutions is necessary to create tailored educational programmes for different youth groups based on their specific needs and levels of understanding. Meanwhile, additional training during the post-graduate period, focused on future healthcare workers, should be provided to enhance their professional skills and equip them to provide accurate information to patients and their families about the purpose and significance of HPV vaccination. This approach would help reduce barriers and dispel doubts among patients.

This study has several strengths and certain limitations. The sample size was relatively small, although the response rate was high, with over 70% of all regularly attending students of the MFNS in the academic year 2023/24 completing the survey. However, the research was conducted at a single medical faculty, and although it is the second largest in the country, it cannot be fully illustrative for other faculties in terms of knowledge levels and correctness of attitudes towards HPV vaccination. Given that few studies have been conducted on this topic among a cohort of students in the Republic of Serbia and the region, generalization of the current findings is challenging. However, data from previous studies share similarities with our results, indicating likely similarities among medical faculty students. The study design allowed for the collection of information from the general student community rather than only those interested in HPV or research. We did not follow up with participants on the decision to receive the HPV vaccine, which might have been useful to estimate the effect of the education. Thus, longitudinal studies are warranted to further explore this issue. Lastly, we cannot exclude the possibility that some students may not have fully understood certain questions in the survey, although researchers were present and available for clarification. Despite the mentioned limitations, we believe that they do not compromise the validity and significance of our research findings.

## 5. Conclusions

Healthcare students, i.e., future healthcare workers, demonstrated a suboptimal percentage of HPV vaccination, certain deficiencies in knowledge and mostly positive attitudes regarding HPV vaccination. Students who had previously attended some form of education on HPV infection and vaccination had significantly higher knowledge compared to those who had not attended such education. Significantly more students would recommend the HPV vaccine to their patients, friends, and family, and they had a significantly higher level of knowledge about HPV and vaccination compared to those who would not recommend it. Finally, the odds of recommending the HPV vaccine increased with each additional correct answer in the knowledge evaluation. Providing thorough, comprehensive education and training for future healthcare providers is vital to prepare them to give clear vaccine recommendations and ensure the best possible health outcomes for their patients. Integrating knowledge and correct attitudes is expected to greatly aid in increasing vaccine coverage and consequently reducing the incidence of HPV infection, cervical cancer and other diseases associated with HPV.

## Figures and Tables

**Table 1:** General socio-demographic characteristics and HPV-related responses of the surveyed population by study programme.

	Total Participants (n = 1760)	Group 1 Study Programmes * (n = 1178)	Group 2 Study Programmes ** (n = 582)	*p*-Value ^#^
Gender, n (%)				
Male	394 (22.39)	288 (24.45)	106 (18.21)	0.003
Female	1366 (77.61)	890 (75.55)	476 (81.79)	
Age, mean (SD)	21.21 (1.98)	21.42 (2.06)	20.78 (1.75)	<0.001
Age category, n (%)				
18–19	397 (22.56)	242 (20.54)	155 (26.63)	<0.001
20–25	1260 (71.59)	847 (71.90)	413 (70.96)	
>25	103 (5.85)	89 (7.56)	14 (2.41)	
Study year, n (%)				
First	416 (23.64)	247 (20.97)	169 (29.04)	NA
Second	383 (21.76)	238 (20.20)	145 (24.91)	
Third	352 (20.00)	223 (18.93)	129 (22.16)	
Fourth	248 (14.09)	156 (13.24)	92 (15.81)	
Fifth	226 (12.84)	179 (15.20)	47 (8.08)	
Sixth	135 (7.67)	135 (11.46)	0	
Study programme with lessons on HPV infection/vaccination (i.e., epidemiology, pediatrics, gynecology classes), n (%)				
Yes	199 (11.31)	199 (16.89)	0	<0.001
No	1561 (88.69)	979 (83.11)	582 (100)	
Have you been vaccinated with the HPV vaccine? n (%)				
No	1572 (89.32)	1054 (89.47)	518 (89.00)	0.799
Yes	171 (9.72)	113 (9.59)	58 (9.97)	
missing	17 (0.97)	11 (0.93)	6 (1.03)	
If you have not been vaccinated, do you plan to get vaccinated against HPV in the near future? n (%)				
No	737 (46.88)	466 (44.21)	271 (52.32)	0.004
Yes	315 (20.04)	223 (21.16)	92 (17.76)	
Yes, if the vaccine is free for my age group	478 (30.41)	342 (32.45)	136 (26.25)	
Missing	42 (2.67)	23 (2.18)	19 (3.67)	
Have you attended any education on HPV infection/vaccination in the past year? n (%)				
Yes	450 (25.57)	348 (29.54)	102 (17.53)	<0.001
No	1288 (73.18)	815 (69.19)	473 (81.27)	
Missing	22 (1.25)	15 (1.27)	7 (1.20)	
In the past month, have you actively sought information regarding HPV infection and vaccination (from your primary care physician, friends, online, etc.)? n (%)				
Yes	260 (14.77)	207 (17.57)	53 (9.11)	<0.001
No	1483 (84.26)	961 (81.58)	522 (89.69)	
Missing	17 (0.97)	10 (0.85)	7 (1.20)	
Do you know anyone who has or has had cervical cancer? n (%)				
Yes	495 (28.13)	328 (27.84)	167 (28.69)	0.756
No	1247 (70.85)	836 (70.97)	411 (70.62)	
Missing	18 (1.02)	14 (1.19)	4 (0.69)	
Self-assessment of knowledge about the HPV vaccine, n (%)				
Sufficient	639 (36.31)	458 (38.88)	181 (31.10)	**0.002**
Insufficient	1072 (60.91)	692 (58.74)	380 (65.29)	
Missing	49 (2.78)	28 (2.38)	21 (3.61)	

Note: Significance levels are bolded for *p* < 0.05. n = number of respondents. NA = not applicable. ^#^ Chi-square test; or Wilcoxon rank-sum test; * group 1 contains study programmes with future health professions that can directly promote HPV vaccination, i.e., study programme of medicine (n = 806), nursing (n = 155) and pharmacy (n = 217); ** group 2 contains future professions of students that can indirectly promote HPV, i.e., not directly working with patients/parents interested in HPV vaccination but can informally promote it, like study programme of dentistry (n = 195), medical rehabilitation (n = 144), radiologic technology (n = 40), and special education and rehabilitation (n = 203).

**Table 2:** Share of students who would recommend the HPV vaccine based on the HPV-related prior experience.

		Would You Recommend the HPV Vaccine to Your Future Patient?			Would You Recommend the HPV Vaccine to Your Family Member/Friend?		
		Yes (n = 1458, 84.62%)	No (n = 265, 15.38%)	*p*-Value *	Yes (n = 1410, 82.46%)	No (n = 300, 17.54%)	*p*-Value *
Did you attend any education on HPV infection/vaccination in the past year?	Yes	401 (89.91)	45 (10.09)	**<0.001**	381 (86.39)	60 (13.61)	**0.012**
	No	1044 (82.99)	214 (17.01)		1015 (81.14)	236 (18.86)	
	missingMissing	13 (68.42)	6 (31.58)		14 (77.78)	4 (22.22)	
Attended study programme with lessons on HPV infection/vaccination (epidemiology, pediatrics, gynecology classes).	Yes	190 (95.96)	8 (4.04)	**<0.001**	181 (93.30)	13 (6.70)	**<0.001**
	No	1268 (83.15)	257 (16.85)		1229 (81.07)	287 (18.93)	
Have you been vaccinated with the HPV vaccine?	Yes	169 (99.41)	1 (0.59)	**<0.001**	167 (99.40)	1 (0.60)	**<0.001**
	No	1276 (82.91)	263 (17.09)		1230 (80.50)	298 (19.50)	
	Missing	13 (92.86)	1 (7.14)		13 (92.86)	1 (7.14)	
In the past month, have you actively sought information regarding HPV infection and vaccination (from your primary care physician, friends, online, etc.)?	Yes	241 (93.05)	18 (6.95)	**<0.001**	235 (91.80)	21 (8.20)	**<0.001**
	No	1203 (83.02)	246 (16.98)		1160 (80.61)	279 (19.39)	
	Missing	14 (93.33)	1 (6.67)		15 (100)	0 (0)	
Do you know anyone who has or has had cervical cancer?	Yes	415 (84.69)	75 (15.31)	0.962	409 (83.64)	80 (16.36)	0.415
	No	1033 (84.60)	188 (15.40)		1001 (81.98)	220 (18.02)	
	Missing	10 (83.33)	2 (16.67)		0	0	
Self-assessment of knowledge about the HPV vaccine	Sufficient	565 (89.88)	65 (10.32)	**<0.001**	542 (87.00)	81 (13.00)	**<0.001**
	Insufficient	855 (81.66)	192 (18.34)		833 (79.87)	210 (20.13)	
	Missing	38 (82.61)	8 (17.39)		35 (79.55)	9 (20.45)	

Note: * Chi-square test. In bold is the statistically significant result at *p* < 0.05.

**Table 3:** Share of correct answers * based on the willingness to recommend the HPV vaccine.

	Total (%)	Would You Recommendthe HPV Vaccine to Your Future Patient?			Would You Recommend the HPV Vaccine to Your Family Member/Friend?		
		Yes (%)	No (%)	*p*-Value ^1^	Yes (%)	No (%)	*p*-Value ^1^
Currently, the HPV vaccine is administered in Serbia, and boys and girls aged 9–19 can receive it free of charge.	69.55	72.5	56.6	**<0.001**	72.62	57.33	**<0.001**
The HPV vaccine is not effective in people over 19 years, so for now, in Serbia, it is only used in persons under 19 years.	55.51	58.02	42.64	**<0.001**	58.23	43.67	**<0.001**
The HPV vaccine also protects against other viral infections.	39.38	40.26	35.85	0.19	40.78	34	0.082
The HPV vaccine is currently given in Serbia in two or three doses, depending on the age of the person.	44.94	47.94	32.08	**<0.001**	47.94	33	**<0.001**
The HPV vaccine can cause HPV infection.	56.93	59.81	43.4	**<0.001**	60.14	43.67	**<0.001**
The HPV vaccine cannot be administered if the person is already sexually active.	57.39	60.36	43.4	**<0.001**	60.71	44	**<0.001**
The HPV vaccine is effective in preventing genital warts in both men and women.	55.57	57.96	44.15	**<0.001**	58.37	43.67	**<0.001**
The HPV vaccine can cause infertility.	53.81	58.57	30.94	**<0.001**	59.22	30.67	**<0.001**
Sexually active individuals should be tested for HPV before starting HPV vaccination.	6.88	7.54	3.4	**<0.001**	7.66	3.67	**<0.001**
The HPV vaccine can prevent cervical cancer.	74.09	77.43	58.87	**<0.001**	77.87	58.67	**<0.001**
After receiving the HPV vaccine, women no longer need to be screened for cervical cancer.	81.59	84.02	70.19	**<0.001**	84.68	69.67	**<0.001**
**Percentage of correct answers per respondent,** **median (IQR)**	54.54 (36.36–72.73)	63.64 (45.46–72.73)	45.46 (27.27–63.64)	**<0.001**	63.64 (45.46–72.73)	45.46 (27.27–63.64)	**<0.001**

Note: * Offered answers were true, false, and I do not know. ^1^ Chi-square test; Wilcoxon rank-sum test. In bold is the statistically significant result at *p* < 0.05.

**Table 4:** Predictors of the willingness of future healthcare workers to recommend HPV vaccine to their patients.

		Unadjusted			Model 1 ^#^		
		OR	95% CI	*p*-Value	aOR	95% CI	*p*-Value
Gender	Male	ref.			-	-	-
	Female	1.17	0.86–1.58	0.325	-	-	-
Age (year)		**1.08**	**1.001–1.15**	**0.048**	-	-	-
Age category	18–19	ref.			-	-	-
	20–24	1.03	0.75–1.40	0.867	-	-	-
	>25	1.57	0.79–3.10	0.196	-	-	-
Study year (year)	first	ref.			ref.		
	second	1.15	0.78–1.70	0.466	1.31	0.86–1.99	0.206
	third	0.76	0.53–1.10	0.149	1.1	0.68–1.77	0.701
	fourth	0.99	0.65–1.51	0.958	1.5	0.82–2.71	0.186
	fifth	**1.71**	**1.04–2.81**	**0.033**	**2.38**	**1.11–5.12**	**0.026**
	sixth	**8.89**	**2.75–28.73**	**<0.001**	**12.53**	**3.15–49.93**	**<0.001**
Study programme *	Group 1	ref.			ref.		
	Group 2	**0.34**	**0.26–0.44**	**<0.001**	**0.38**	**0.26–0.54**	**<0.001**
Study programme with lessons on HPV infection/vaccination	No	**ref.**			**ref.**		
	Yes	**4.81**	**2.34–9.89**	**<0.001**	**4.25**	**2.02–8.95**	**<0.001**
Have you been vaccinated with the HPV vaccine?	No	ref.			-	-	-
	Yes	**34.83**	**4.86–249.83**	**<0.001**	**-**	**-**	**-**
Do you plan to get vaccinated against HPV in the near future?	No	ref.			ref.		
	Yes	**16.06**	**8.13–31.73**	**<0.001**	**17.19**	**8.63–34.23**	**<0.001**
	Yes, if the vaccine is free of charge for my age group	**11.12**	**6.85–18.06**	**<0.001**	**12.01**	**7.34–19.66**	**<0.001**
Have you attended any education on HPV infection/vaccination in the past year?	Yes	**1.83**	**1.30–2.57**	**0.001**	**1.67**	**1.17–2.37**	**0.004**
	No	ref.			ref.		
In the past month, have you actively sought information regarding HPV infection and vaccination (from your primary care physician, friends, online, etc.)?	Yes	**2.74**	**1.66–4.51**	**<0.001**	**2.48**	**1.50–4.11**	**<0.001**
	No	ref.			ref.		
Do you know anyone who has or has had cervical cancer?	Yes	1.01	0.75–1.35	0.962	0.99	0.74–1.35	0.984
	No	ref.			ref.		
Self-assessment of knowledge about the HPV vaccine	Sufficient	**1.95**	**1.45–2.64**	**<0.001**	**1.62**	**1.19–2.21**	**0.002**
	Insufficient	ref.			ref.		
Number of correct answers on the knowledge evaluation		**1.23**	**1.17–1.28**	**<0.001**	**1.19**	**1.14–1.25**	**<0.001**

Note: * Group 1 contains study programmes with future health professions that can directly promote HPV vaccination, i.e., the study programme of medicine (n = 806), nursing (n = 155) and pharmacy (n = 217). Group 2 contains future professions of students that can indirectly promote HPV, i.e., not directly working with patients/parents interested in HPV vaccination but can informally promote it, like study programme of dentistry (n = 195), medical rehabilitation (n = 144), radiologic technology (n = 40), and special education and rehabilitation (n = 203). aOR = adjusted odds ratio. ^#^ Model 1 adjusted for gender, age, study programme, and previous HPV vaccination. In bold is the statistically significant result at *p* < 0.05.

## Data Availability

The original contributions presented in this study are included in the article/supplementary materials. Further inquiries can be directed to the corresponding author(s).

## Supplementary Materials

The following supporting information can be downloaded at https://www.mdpi.com/article/10.3390/vaccines13010011/s1. Table S1: Checklist for Reporting Of Survey Studies (CROSS); Table S2: Survey questionnaire translated into English; Table S3: The HPV-related prior experience of students based on the attendance of classes covering the topic of HPV; Figure S1: Prevalence of attitudes about the HPV vaccine among students of all study programmes and stratified by attending previous education on HPV; Figure S2: Prevalence of attitudes about the HPV vaccine among students based on the study programme covering classes with HPV-related topic.

## References

[1] World Health Organization (2017). Human papillomavirus vaccines. WHO position paper. Wkly Epidemiol. Rec..

[2] Illah O., Olaitan A. (2023). Updates on HPV Vaccination. Diagnostics.

[3] Yousefi Z., Aria H., Ghaedrahmati F., Bakhtiari T., Azizi M., Bastan R., Hosseini R., Eskandari N. (2022). An Update on Human Papilloma Virus Vaccines: History, Types, Protection, and Efficacy. Front. Immunol..

[4] de Oliveira C.M., Fregnani J.H., Villa L.L. (2019). HPV Vaccine: Updates and Highlights. Acta Cytol..

[5] Murillo R., Ordóñez- Reyes C. (2019). Human Papillomavirus (HPV) Vaccination: From Clinical Studies to Immunization Programs. Int. J. Gynecol. Cancer.

[6] Vuković V., Štrbac M., Ristić M., Skočibušić S., Cilović-Lagarija Š., Aćimović J., Šiljak S., Živković Perišić S., Nikolić N., Ljubičić S. (2024). Relationship Between Population Density, Availability of Gynecological Services, and Cervical Cancer Incidence and Mortality Across Administrative Units in Serbia and Bosnia and Herzegovina During 2016–2020. Medicina.

[7] Gradski Zavod za Javno Zdravlje Beograd—Vakcina Protiv Oboljenja Izazvanih Humanim Papilomavirusima (HPV). https://www.zdravlje.org.rs/index.php/aktuelne-vesti/1105-vakcina-protiv-oboljenja-izazvanih-humanim-papiloma-virusima-hpv.

[8] Štrbac M., Joksimović M., Vuković V., Ristić M., Lončarević G., Kanazir M., Nikolić N., Pustahija T., Rajčević S., Ljubičić S. (2024). Overview of the Implementation of the First Year of Immunization against Human Papillomavirus across Different Administrative Units in Serbia and Montenegro. Vaccines.

[9] Miraglia del Giudice G., Sansone V., Della Polla G., Angelillo I.F. (2024). Understanding the Reasons for Receiving HPV Vaccination among Eligible Adults in Italy. Vaccines.

[10] Escoffery C., Petagna C., Agnone C., Perez S., Saber L.B., Ryan G., Dhir M., Sekar S., Yeager K.A., Biddell C.B. (2023). A systematic review of interventions to promote HPV vaccination globally. BMC Public Health.

[11] Oketch S.Y., Ochomo E.O., Orwa J.A., Mayieka L.M., Abdullahi L.H. (2023). Communication strategies to improve human papillomavirus (HPV) immunisation uptake among adolescents in sub-Saharan Africa: A systematic review and meta-analysis. BMJ Open.

[12] Wu H., Tong X., Wang L., Huang Y., Zhang L. (2023). HPV vaccine information, knowledge, attitude, and recommendation intention among male college students in China. Hum. Vaccines Immunother..

[13] Daniel C.L., McLendon L., Green C.L., Anderson K.J., Pierce J.Y., Perkins A., Beasley M. (2021). HPV and HPV vaccination knowledge and attitudes among medical students in Alabama. J. Cancer Educ..

[14] Sharma A., Minh Duc N.T., Luu Lam Thang T., Nam N.H., Ng S.J., Abbas K.S., Huy N.T., Marušić A., Paul C.L., Kwok J. (2021). A consensus-based checklist for reporting of survey studies (CROSS). J. Gen. Intern. Med..

[15] Textor J., Van der Zander B., Gilthorpe M.S., Liśkiewicz M., Ellison G.T. (2016). Robust causal inference using directed acyclic graphs: The R package ‘dagitty’. Int. J. Epidemiol..

[16] Kutz J.M., Rausche P., Gheit T., Puradiredja D.I., Fusco D. (2023). Barriers and facilitators of HPV vaccination in sub-saharan Africa: A systematic review. BMC Public Health.

[17] Sackey M.E., Markey K., Grealish A. (2022). Healthcare professional’s promotional strategies in improving Human papillomavirus (HPV) vaccination uptake in adolescents: A systematic review. Vaccine.

[18] Dufour L., Carrouel F., Dussart C. (2023). Human Papillomaviruses in Adolescents: Knowledge, Attitudes, and Practices of Pharmacists Regarding Virus and Vaccination in France. Viruses.

[19] Lee H.Y., Daniel C.L., Wang K., McLendon L., Coyne-Beasley T. (2023). HPV Vaccination among College Students in the South: The Role of HPV Knowledge on Vaccine Initiation and Completion. Asian Pac. J. Cancer Prev. APJCP.

[20] Kellogg C., Shu J., Arroyo A., Dinh N.T., Wade N., Sanchez E., Equils O. (2019). A significant portion of college students are not aware of HPV disease and HPV vaccine recommendations. Hum. Vaccines Immunother..

[21] Korkmaz D., Turunç H.N., Özarslan Y.A., Yıldırım Ü., Büyükarmutcu Y., Dağlı S., Manavlı B. (2023). Assessment of HPV Vaccine Knowledge Levels Among Medical Faculty Students: A Comprehensive Examination in the Turkish Context: HPV Vaccine Knowledge among Turkish Medical Students. Med. Sci. Discov..

[22] Leung J.T., Law C.K. (2018). Revisiting knowledge, attitudes and practice (KAP) on human papillomavirus (HPV) vaccination among female university students in Hong Kong. Hum. Vaccines Immunother..

[23] Alkhaldi R.O., Alzahrani H.A., Metwally L.A. (2023). Awareness level about cervical cancer, human papillomavirus (HPV) and corresponding vaccine among women living in the Western Region of Saudi Arabia. Cureus.

[24] Gilkey M.B., Calo W.A., Moss J.L., Shah P.D., Marciniak M.W., Brewer N.T. (2016). Provider communication and HPV vaccination: The impact of recommendation quality. Vaccine.

[25] Gilkey M.B., Grabert B.K., Malo T.L., Hall M.E., Brewer N.T. (2020). Physicians’ rhetorical strategies for motivating HPV vaccination. Soc. Sci. Med..

[26] Yin G., Zhang Y., Chen C., Ren H., Guo B., Zhang M. (2021). Have you ever heard of Human Papillomavirus (HPV) vaccine? The awareness of HPV vaccine for college students in China based on meta-analysis. Hum. Vaccines Immunother..

[27] Pourmohsen M., Simbar M., Nahidi F., Fakor F., Majd H.A. (2018). HPV risk factors and prevention behaviours: A review. J. Clin. Diagn. Res..

[28] Şahin H., Özerdoğan Ö., Duran M.N. (2020). Knowledge, attitudes, and behaviors of medical students regarding HPV and HPV vaccine. Fam. Pract. Palliat. Care.

[29] Gilkey M.B., Moss J.L., Coyne-Beasley T., Hall M.E., Shah P.D., Brewer N.T. (2015). Physician communication about adolescent vaccination: How is human papillomavirus vaccine different?. Prev. Med..

[30] Hswen Y., Gilkey M.B., Rimer B.K., Brewer N.T. (2017). Improving physician recommendations for human papillomavirus vaccination: The role of professional organizations. Sex. Transm. Dis..

[31] Lake P.W., Kasting M.L., Christy S.M., Vadaparampil S.T. (2019). Provider perspectives on multilevel barriers to HPV vaccination. Hum. Vaccines Immunother..

[32] CDC (2019). HPV Vaccine Information For Young Women. https://www.cdc.gov/std/hpv/stdfact-hpv-vaccine-young-women.htm.

[33] Lee H.Y., Lee J., Henning-Smith Choi J. (2017). HPV literacy and its link to initiation and completion of HPV vaccine among young adults in Minnesota. Public Health.

[34] Hopkins T.G., Wood N.J., West R.M., Darling J.C. (2009). UK health professionals’ attitudes and knowledge regarding Human Papillomavirus (HPV) vaccination: A West Yorkshire Study. J. Paediatr. Child Health.

[35] Lin C., Mullen J., Smith D., Kotarba M., Kaplan S.J., Tu P. (2021). Healthcare providers’ vaccine perceptions, hesitancy, and recommendation to patients: A systematic review. Vaccines.

[36] Rosenthal S.L., Weiss T.W., Zimet G.D., Ma L., Good M.B., Vichnin M.D. (2011). Predictors of HPV vaccine uptake among women aged 19–26: Importance of a physician’s recommendation. Vaccine.

[37] Liu A., Ho F.K., Chan L.K., Ng J.Y., Li S.L., Chan G.C., Leung T.F., Ip P. (2018). Chinese medical students’ knowledge, attitude and practice towards human papillomavirus vaccination and their intention to recommend the vaccine. J. Paediatr. Child Health.

[38] Gollu A.N., Gore C.A. (2021). Knowledge, awareness and attitude of medical students regarding HPV infection and HPV vaccination. Asian Pac. J. Cancer Care.

[39] Yam P.W.A., Lam P.L., Chan T.K., Chau W.K., Hsu L.M., Lim M., Lo C.H., Siu L., Tang H.F., Tong A.M.J.M. (2017). A Cross Sectional Study on Knowledge, Attitude and Practice related to Human Papillomavirus Vaccination for Cervical Cancer Prevention between Medical and Non-Medical Students in Hong Kong. Asian Pac. J. Cancer Prev..

[40] Monteiro D.L.M., Brollo L.C.S., Souza T.P., Santos G.R., Correa T., da Costa J.T., de Oliveira M.A.P., Trajano A.J.B. (2018). Knowledge on the HPV vaccine among university students. Rev. Inst. Med. Trop. Sao Paulo..

