# Supplementary material for: Knowledge and Attitudes Regarding Human Papillomavirus Vaccination Among Future Healthcare Workers in Serbia
Source: Vaccines (Basel). 2024 Dec 26;13(1):11. doi: 10.3390/vaccines13010011 (PMC11768562; doi:10.3390/vaccines13010011)
Supplement: Supplementary file 1 [file vaccines-13-00011-s001.zip › vaccines-3346359-supplementary.pdf]

Table S1. Checklist for Reporting Of Survey Studies (CROSS)

Based on the manuscript "Knowledge and Attitudes regarding Human Papillomavirus Vaccination among Future Healthcare Workers in Serbia"

Section/Topic	Item	Item Description	Reported on Page #
**Title and Abstract**			
Title and Abstract	1a	State the word "survey" along with a commonly used term in title or abstract to introduce the study's design.	1 (Lines 15-16)
	1b	Provide an informative summary in the abstract, covering background, objectives, methods, findings/results, interpretation/discussion and conclusions.	1 (Lines 11-30)
**Introduction**			
Background Purpose/aim	2	Provide a background about the rationale of study, what has been previously done, and why this survey is needed.	1-2 (Lines 34-80)
	3	Identify specific purposes, aims, goals, or objectives of the study.	2 (Lines 78-80)
**Methods**			
Study Design	4	Specify the study design in the methods section with a commonly used term (e.g., cross-sectional or longitudinal).	2 (Line 83)
Data Collection Methods	5a	Describe the questionnaire (e.g., number of sections, number of questions, number and names of instruments used).	3-4(Lines 129-139)
	5b	Describe all questionnaire instruments used in the survey to measure particular concepts.	3-4 (Lines 129-139)
	5c	Report target population, reported validity and reliability information, scoring/classification procedure, and reference links (if any).	2 (Lines 83-89)
	5d	Provide information on pretesting of thequestionnaire, if performed, including method,number, demographics of participants, andsimilarity to the target population.	3 (Lines 127-129)
Sample Characteristics	6a	Describe the study population (i.e., background, locations, eligibility criteria for participant inclusion in survey, exclusion criteria).	2-3 (Lines 83-106)
	6b	Describe the sampling techniques used (e.g., single stage, multistage sampling, simple randomsampling). Specify the locations if clusteredsampling was applied.	3 (Lines 120-127)
	6c	Provide information on sample size, along with details of sample size calculation.	2-3 (Lines 92-112)
	6d	Describe how representative the sample is of the study population, particularly for	2-3 (Lines 91-102)

		population-based surveys.	
Survey Administration	7a	Provide information on modes of questionnaire administration, including type and number of contacts, and location of survey administration.	3 (Lines 120-127)
	7b	Provide information on survey's time frame, including periods of recruitment, exposure, andfollow-up days.	3 (Lines 120-127)
	7c	Provide information on the entry process:<br>–>For non-web-based surveys, provide approaches to minimize human error indata entry.<br>–>For web-based surveys, provideapproaches to prevent "multipleparticipation"of participants.	4 (Lines 141-154)
Study preparation	8	Describe any preparation process before conducting the survey, e.g., interviewers' training process or survey advertisement.	N/A
Ethical Considerations	9a	Provide information on ethical approval, including informed consent, institutional review board (IRB) approval, Helsinki declaration, and good clinical practice (GCP).	4 (Lines 140-145)
	9b	Report information about anonymity and confidentiality mechanisms.	4 (Lines 141-154)
Statistical Analysis	10a	Describe statistical methods and analyticalapproach. Report the software used for dataanalysis.	4 (Lines 147-169)
	10b	Report any modification of variables used in the analysis, along with reference (ifavailable).	N/A
	10c	Report details about how missing data was handled. Include rate of missing items, missing data mechanism (i.e., missing completely at random [MCAR], missing atrandom [MAR] or missing not at random [MNAR]) and methods used to deal withmissing data (e.g., multiple imputation).	N/A
	10d	State how non-response error was addressed.	N/A
	10e	For longitudinal surveys, state how loss to follow-up was addressed.	N/A
	10f	Indicate whether any methods such as weighting of items or propensity scoreshave been used to adjust for non-representativeness of the sample.	N/A
	10g	Describe any sensitivity analysis conducted.	N/A
**Results**			
Respondent characteristics	11a	Report numbers of individuals at each stage of the study. Consider using a flowdiagram, if possible.	5-6 (Table 1.)
	11b	Provide reasons for non-participation at each stage, if possible.	N/A

	11c	Report response rate, present the definition ofresponse rate or the formula usedto calculate response rate.	N/A
	11d	Provide information to define how unique visitors are determined. Report numberof unique visitors along with relevant proportions (e.g., view proportion, participation proportion, completion proportion).	N/A
Descriptive results	12	Provide characteristics of study participants, as wellas information on potentialconfounders and assessed outcomes.	5-6 (Table 1.)
Main findings	13a	Give unadjusted estimates and, if applicable, confounder-adjusted estimates alongwith 95% confidence intervals and p-values.	Descriptive analysis only
	13b	For multivariable analysis, provide information on the model building process,model fit statistics, and model assumptions (as appropriate).	N/A
	13c	Provide details about any sensitivity analysis performed. If there are considerableamount of missing data, report sensitivity analyses comparing the results ofcomplete cases with that of theimputed dataset (if possible).	No sensitivity analysis performed
**Discussion**			
Limitations	14	Discuss the limitations of the study, considering sources of potential biases andimprecisions, such as non-representativeness of sample, study design, importantuncontrolled confounders.	15 (Lines 484-501)
Interpretations	15	Give a cautious overall interpretation of results, based on potential biases andimprecisions and suggest areas for future research.	12-15
Generalizability	16	Discuss the external validity of the results.	15
**Other sections**			
Role of funding source	17	State whether any funding organization has had any roles in the survey's design, implementation, and analysis.	15 (Lines 531)
Conflict of interest	18	Declare any potential conflict of interest.	16 (Lines 542)
Acknowledgements	19	Provide names of organizations/persons that areacknowledged along with theircontribution to the research.	16 (Lines 539-541)

Table S2. Survey questionnaire translated into English

Date__________
ID code________

Knowledge and Attitudes about HPV Infection, Prevention Methods, and HPV Vaccination among Medical Faculty Students: A Cross-Sectional Study

Please carefully read the following questions and respond by selecting the appropriate answers or writing your responses where indicated.

1. **Gender**: ☐ Male ☐ Female
2. **Age (in years)**: ____________
3. **Study Program**: ☐ Medicine ☐ Dentistry ☐ Pharmacy ☐ Nursing ☐ (other) ______________
4. **Current Year of Study (please specify)**: _________
5. **Place of Residence during Childhood**: ______________________
6. **Number of Siblings**:
   a) Younger than you: ______
   b) Older than you: _______
7. **Have you been vaccinated with the HPV vaccine?**
   a) No ☐ b) Yes, I have received: ☐ Cervarix ☐ Gardasil4 ☐ Gardasil9
   If NO, do you plan to get vaccinated in the near future?
   a) No ☐ b) Yes ☐ c) Yes, if the vaccine is free for my age group ☐
8. **In the past year, have you attended any educational session on HPV infection/vaccine?**
   a) Yes ☐ b) No ☐
   If YES, please specify: ☐ Lecture as part of studies ☐ Education by a doctor (epidemiologist, pediatrician, gynecologist) ☐ Peer education ☐ Other (please specify) _______________
9. **Have you actively sought information about HPV infection and vaccination in the past month (from a healthcare provider, friends, the internet, etc.)?**
   a) Yes ☐ b) No ☐
10. **How do you assess your knowledge about:**
   - HPV infection: ☐ Sufficient ☐ Insufficient
   - Methods of preventing HPV infection: ☐ Sufficient ☐ Insufficient
   - HPV vaccine: ☐ Sufficient ☐ Insufficient
11. **Would you recommend the HPV vaccine to your future patient?**
   a) Yes ☐ b) No ☐
12. **Would you recommend the HPV vaccine to a family member/friend?**
   a) Yes ☐ b) No ☐
13. **Do you know anyone who has had cervical cancer?**
   a) Yes ☐ b) No ☐

14. Please mark the appropriate response (True/False/Don't know) for the following statements about HPV vaccine:

- The HPV vaccine is currently available in Serbia and is free for boys and girls aged 9-19. ☐ True ☐ False ☐ Don't know
- The HPV vaccine is not effective in people older than 19, so it is only available for people under 19 in Serbia. ☐ True ☐ False ☐ Don't know
- The HPV vaccine also protects against other viral infections. ☐ True ☐ False ☐ Don't know
- The HPV vaccine in Serbia is given in two or three doses, depending on the person's age. ☐ True ☐ False ☐ Don't know
- The HPV vaccine can cause an HPV infection. ☐ True ☐ False ☐ Don't know
- The HPV vaccine cannot be given to someone who is already sexually active. ☐ True ☐ False ☐ Don't know
- The HPV vaccine is effective in preventing genital warts in both men and women. ☐ True ☐ False ☐ Don't know
- The HPV vaccine can cause infertility. ☐ True ☐ False ☐ Don't know
- Sexually active people should be tested for HPV before starting vaccination. ☐ True ☐ False ☐ Don't know
- The HPV vaccine can prevent cervical cancer. ☐ True ☐ False ☐ Don't know
- After receiving the HPV vaccine, women no longer need cervical cancer screening. ☐ True ☐ False ☐ Don't know

20. Please indicate your level of agreement with the following statements (scale 1-5, where 1 = strongly disagree and 5 = strongly agree):

- I believe only women should be vaccinated against HPV. ☐ 1 ☐ 2 ☐ 3 ☐ 4 ☐ 5
- I believe the HPV vaccine is not completely safe for health. ☐ 1 ☐ 2 ☐ 3 ☐ 4 ☐ 5
- I don't trust any vaccine, including the HPV vaccine. ☐ 1 ☐ 2 ☐ 3 ☐ 4 ☐ 5
- I believe the HPV vaccine is unnecessary for people who don't change sexual partners frequently. ☐ 1 ☐ 2 ☐ 3 ☐ 4 ☐ 5
- Condoms provide complete protection against HPV, so there is no need for vaccination if protection is used during sexual intercourse. ☐ 1 ☐ 2 ☐ 3 ☐ 4 ☐ 5
- I believe HPV vaccination in teenagers increases the risk of unprotected sexual activity. ☐ 1 ☐ 2 ☐ 3 ☐ 4 ☐ 5
- I would get vaccinated against HPV, but the vaccine is not free for my age group. ☐ 1 ☐ 2 ☐ 3 ☐ 4 ☐ 5
- I believe the HPV vaccine is not sufficiently researched because it has only recently been introduced in Serbia. ☐ 1 ☐ 2 ☐ 3 ☐ 4 ☐ 5
- I believe it is essential to organize additional educational sessions about HPV vaccination. ☐ 1 ☐ 2 ☐ 3 ☐ 4 ☐ 5

Thank you for your participation!

Table S3. The HPV-related prior experience of students based on the attendance of classes covering HPV topic.

	Students without HPV-related classes (n=1561, 88.69%)[1]	Students with HPV-related classes (n=199, 11.31%)[2]	p-value
**If you have not been vaccinated, do you plan to get vaccinated against HPV in the near future? n=1572 (89.32%)**			
No	637 (45.99)	100 (53.48)	**0.035**
Yes	290 (20.94)	25 (13.37)	
Yes, if the vaccine is free for my age group	418 (30.18)	60 (32.09)	
missing	40 (2.89)	2 (1.07)	
**In the past month, have you actively sought information regarding HPV infection and vaccination (from your primary care physician, friends, online, etc.)?**			
Yes	207 (13.26)	53 (26.63)	**<0.001**
No	1338 (85.71)	145 (72.86)	
missing	16 (1.02)	1 (0.50)	
**Self-assessment of knowledge about the HPV vaccine**			
Sufficient	518 (33.18)	121 (60.80)	**<0.001**
Insufficient	1000 (64.06)	72 (36.18)	
missing	43 (2.75)	6 (3.02)	
**Would you recommend the HPV vaccine to your future patient?**			
yes	1268 (81.23)	190 (95.48)	**<0.001**
no	257 (16.46)	8 (4.02)	
missing	36 (2.31)	1 (0.50)	
**Would you recommend the HPV vaccine to your family member/friend?**			
yes	1229 (78.73)	181 (90.95)	**<0.001**
no	287 (18.39)	13 (6.53)	
missing	45 (2.88)	5 (2.51)	

Notes: [1]first to fifth year medicine students, first and second year nursing students, all students of study programmes in pharmacy, dentistry, medical rehabilitation, radiological technology, and special education and rehabilitation. [2]sixth year medicine students, third- and fourth-year nursing students.

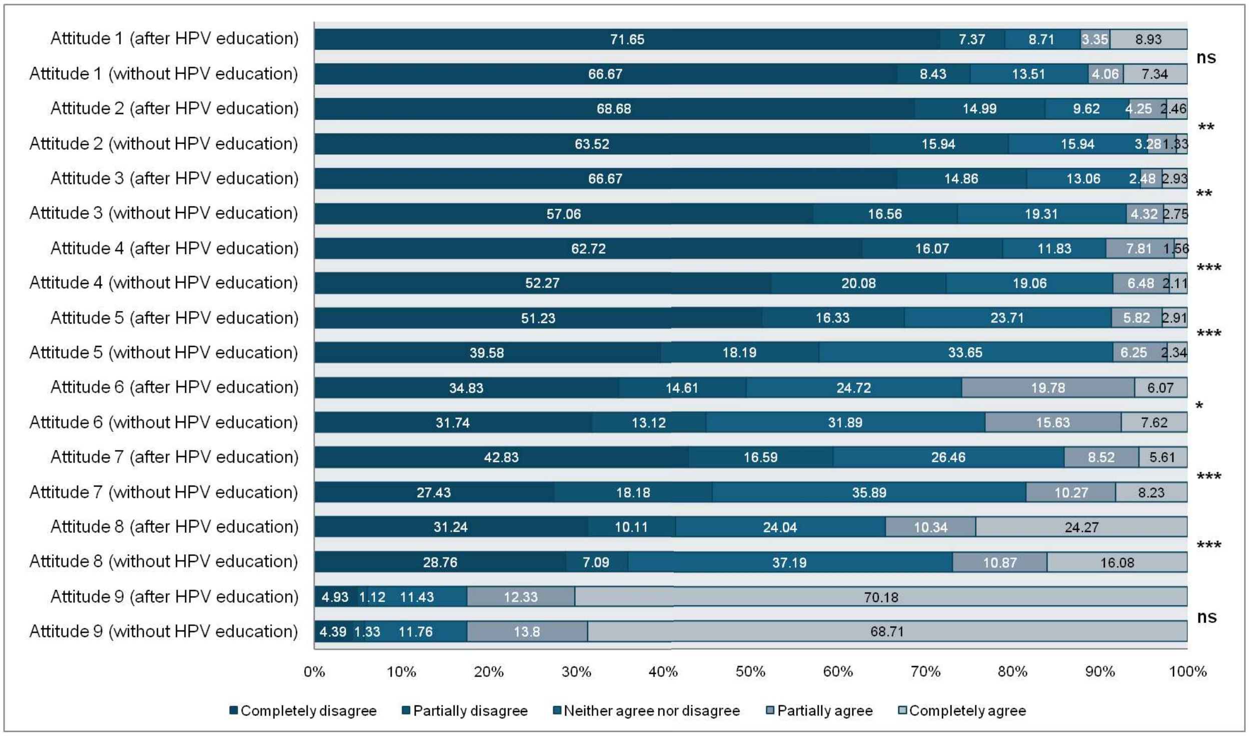

Figure S1. Prevalence of attitudes about the HPV vaccine among students of all study programs and stratified by attending previous education on HPV. Note: Attitude 1: I believe that only females should be vaccinated against HPV.; Attitude 2: I believe that the HPV vaccine is not necessary for people who do not often change sexual partners.; Attitude 3: I do not trust any vaccine, including the one against HPV.; Attitude 4: Condoms provide complete protection against HPV, so there is no need for people to be vaccinated if they use protection during sex.; Attitude 5: I believe that the HPV vaccine is not completely safe for health.; Attitude 6: I believe that HPV vaccination in teenagers increases the risk of unprotected sex.; Attitude 7: I believe that the HPV vaccine has not yet been sufficiently tested because it has only recently been introduced in Serbia.; Attitude 8: I would get vaccinated against HPV, but the vaccine is not free of charge for my age.; Attitude 9: I believe that it is necessary to organize additional education about HPV vaccination. Using Chi-square test: *$p<0.05$; **$p<0.01$; ***$p<0.001$; ns=not statistically significant.

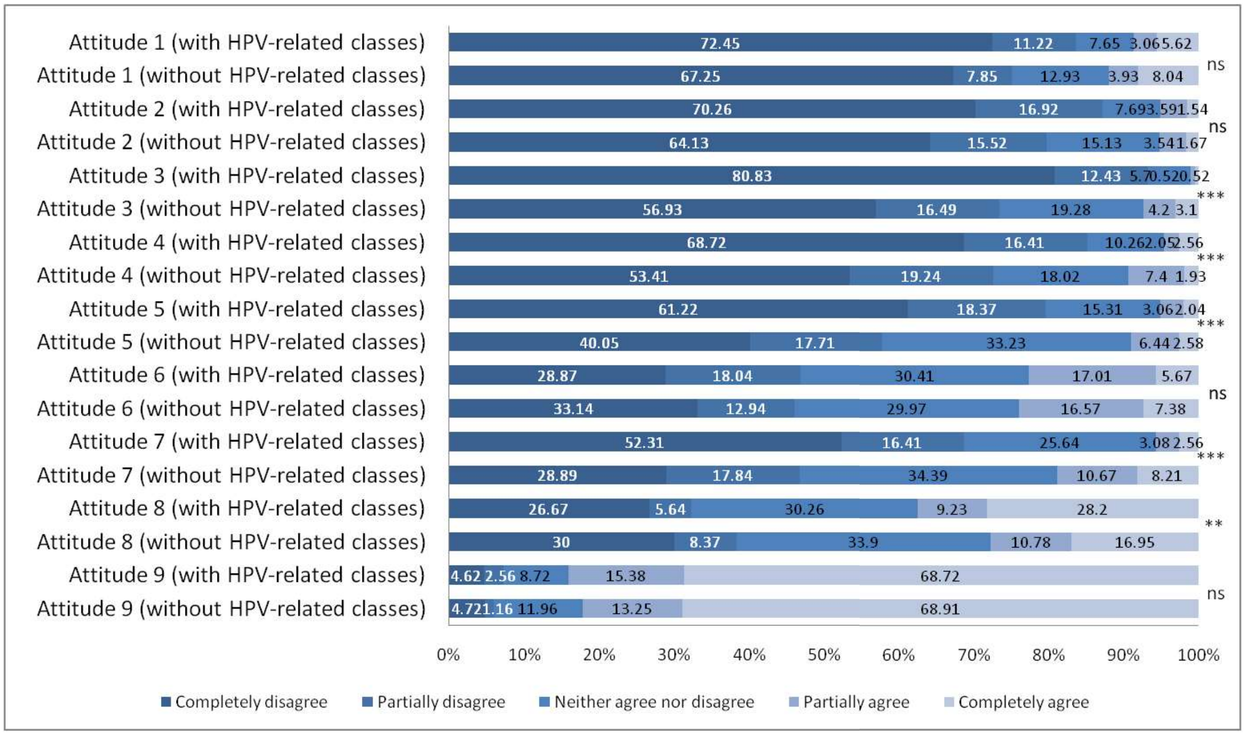

Figure S2. Prevalence of attitudes about the HPV vaccine among students based on the study programme covering classes with HPV-related topic. Note: Attitude 1: I believe that only females should be vaccinated against HPV.; Attitude 2: I believe that the HPV vaccine is not necessary for people who do not often change sexual partners.; Attitude 3: I do not trust any vaccine, including the one against HPV.; Attitude 4: Condoms provide complete protection against HPV, so there is no need for people to be vaccinated if they use protection during sex.; Attitude 5: I believe that the HPV vaccine is not completely safe for health.; Attitude 6: I believe that HPV vaccination in teenagers increases the risk of unprotected sex.; Attitude 7: I believe that the HPV vaccine has not yet been sufficiently tested because it has only recently been introduced in Serbia.; Attitude 8: I would get vaccinated against HPV, but the vaccine is not free of charge for my age.; Attitude 9: I believe that it is necessary to organize additional education about HPV vaccination. Using Chi-square test: *$p<0.05$; **$p<0.01$; ***$p<0.001$; ns=not statistically significant.
